# Detecting Body Fat–A Weighty Problem BMI versus Subcutaneous Fat Patterns in Athletes and Non-Athletes

**DOI:** 10.1371/journal.pone.0072002

**Published:** 2013-08-26

**Authors:** Renate Kruschitz, Sandra J. Wallner-Liebmann, Michael J. Hamlin, Maximilian Moser, Bernhard Ludvik, Wolfgang J. Schnedl, Erwin Tafeit

**Affiliations:** 1 Medical University Vienna, Clinic for Internal Medicine III, Department of Endocrinology and Metabolism, Vienna, Austria; 2 Medical University Graz, Inst. of Pathophysiology and Immunology, Graz, Austria; 3 SIPCAN Special Institute for Preventive Cardiology and Nutrition, Salzburg, Austria; 4 Lincoln University, Environment Society and Design Faculty, Christchurch, New Zealand; 5 Medical University Graz, Inst. of Physiology, Graz, Austria; 6 Practice for General Internal Medicine, Bruck/Mur, Austria; 7 Medical University Graz, Inst. of Physiological Chemistry, Graz, Austria; St. Joseph’s Hospital and Medical Center, United States of America

## Abstract

We aimed to describe the relationship between BMI and the subcutaneous adipose tissue topography within young athletes and non-athletic controls, to comparatively evaluate the diagnostic powers of subcutaneous adipose tissue thicknesses at different body sites, furthermore to explore appropriate cut-offs to discriminate between athletes and controls. Measurements were determined in 64 males and 42 females, who were subsequently separated into two even groups (athletes and non-athletes). The optical device LIPOMETER was applied at standardised body sites to measure the thickness of subcutaneous adipose tissue layers. To calculate the power of the different body sites and the BMI to discriminate between athletes and non-athletes, receiver operating characteristic curve analysis was performed. In men, the neck (optimal cut-off value 2.3 mm) and trunk (optimal cut-off value 15.5 mm) provided the strongest discrimination power: with 90.6% (58 of 64) of the subjects being correctly classified into athletes or non-athletes. Discrimination power of the BMI values was 64.1% (41 of 64 were correctly classified). In women, the upper back (optimal cut-off value 3.3 mm) and arms (optimal cut-off value 15.9 mm) provided the strongest discrimination power with 88.1% (37 of 42 being correctly classified). When using BMI to discriminate between athletes and non-athletes only 52.4% (22 of 42) were correctly classified. These results suggest that compared to BMI levels, subcutaneous fat patterns are a more accurate way of discriminating between athletes and non-athletes. In particular the neck and the trunk compartment in men and the upper back and arms compartment in women, were the best sites to discriminate between young athletes and non-athletes on the basis of their fat patterns.

## Introduction

Since James S. Garrow proposed the body mass index (BMI, kg/m^2^) as a measure of fatness in 1985 [1], its use in science and within clinical practice has risen exponentially over the years. Especially in sports science the BMI and assessment of body fat to determine optimal body weight has increased [2]. Body weight and body composition are important performance factors in many sports [3]. A centralized subcutaneous fat distribution has been associated with decreased aerobic capacity in men [4]. In both athletic and non-athletic populations the estimates of body composition characteristics are used to identify health status [5]. Nevertheless the use of body weight by itself and/or BMI has been criticized, particularly in athletic populations [6]–[8]. The BMI indicates a somewhat stronger yet still moderate association with body fat and disease risk compared to estimates based on stature and body mass [9], [10]. Although BMI is correlated (r = 0.60–0.82) with percentage total body fat (TBF%) [11], there is a lack of research regarding the usefulness of BMI as a surrogate for TBF%, especially in young adults and athletes. The BMI does not discriminate between the different components of the body and cannot describe the fat distribution over the body. Individuals with high fat-free mass (FFM) relative to height, like athletes and younger adults, might have a high BMI but they are not necessarily obese [2], [6], [8].

In general, there is little consensus on the use of body fat percentage criteria to define obesity or excess body fat levels [12]. The American College of Sports Medicine (ACSM) [13] recommended on the basis of data reported by Gallagher and colleagues [14] a TBF% over 33% in women and 20% in men as acceptable cut-points for overfatness, corresponding to a BMI of 25 kg/m^2^, in athletes. Recently published TBF% cut-offs from Heo et al. [15] which are comparable with those of Gallagher et al. [14] tend to be higher, especially in younger groups regardless of age, sex and ethnicity. Heo et al. [15] assume that 35–37% TBF% in women and 23–25% TBF% in men corresponds to a BMI of 25 kg/m^2^ in young African Americans and white adults (aged 18–29).

Compared with the general adult population, the influence of a large muscle mass on BMI in athletes and young adults misclassify these individuals as overweight and obese [16]. Probably more important in assessing the health risks of excessive fat stores is the distribution of fat over the body [17]. Therefore, the use of TBF% and subcutaneous fat patterns may be more effective than BMI in assessing fatness and obesity in physically active individuals and young adults.

The computerized optical device named the Lipometer (Moeller Messtechnik, Graz, EU patent number 0516251) allows a non-invasive, quick, precise and safe determination of the thickness of subcutaneous adipose tissue (SAT) layers at any chosen site of the human body.

As far as we know there has been no study that has assessed the relationship between BMI and SAT-Top in young athletes and non-athletes. Therefore the purpose of this study was to prove our hypothesis that compared to BMI levels the subcutaneous fat patterns are a better screening tool to characterize fatness in athletes compared to non-athletes. A secondary aim of this study was to provide appropriate subcutaneous adipose tissue measuring points and cut-offs that allow in a quick and precise way to discriminate between athletes and non-athletic controls.

## Subjects and Methods

### Subjects

In this cross-sectional study the age, height, weight, BMI and SAT-Top were determined in 64 men (32 athletes and 32 non-active controls matched in age, height, weight and BMI) and 42 women (21 athletes and 21 non-active controls with comparable age and height). The female athletes had a significantly higher weight and BMI compared to the control females. Subjects wore light clothing (e.g. shorts and a light top) and no shoes during the measurements. Standing height was measured to the nearest 0.1 cm using a portable calibrated stadiometer (SECA®-220, Hamburg, Germany). Body mass was measured to the nearest 0.01 kg using calibrated electronic scales (Soehnle® 7700, Murrhardt, Germany) and BMI was calculated as body mass (kg) divided by height (m) squared. To record the extent of training and competition load in individuals, structured questionnaires were used from which training volume in kilometres and hours per week was calculated. Descriptive characteristics of the groups are presented in Table 1 and 2.

**Table 1:** Descriptive statistics of the two male groups.

Personal parameters	Male non-athletes (n = 32)	Male athletes (n = 32)	Significance of differences^1^
Age (y)	25.8±5.6 (22.1–27.7)	23.0±13.2 (17.8–31.0)	n.s.^2^
Height (m)	1.80±0.1 (1.75–1.82)	1.8±0.1 (1.75–1.84)	n.s.^3^
Weight (kg)	72.3±8.7 (66.3–75.0)	72.0±8.5 (66.3–74.8)	n.s.^3^
BMI (kg/m^2^)	22.4±1.4 (21.6–23.0)	21.8±2.3 (20.7–23.0)	n.s.^3^
**SAT-Top** 4			
Neck	3.7±3.7 (2.5–6.2)	1.2±0.6 (1.0–1.6)	p<0.001
Triceps	4.9±3.0 (3.5–6.5)	2.1±1.9 (1.5–3.4)	p<0.001
Biceps	3.0±1.6 (2.1–3.7)	1.5±0.6 (1.2–1.8)	p<0.001
Upper back	3.6±2.3 (2.5–4.8)	1.5±1.0 (1.1–2.1)	p<0.001
Front chest	3.8±2.9 (2.8–5.7)	1.8±1.2 (1.3–2.5)	p<0.001
Lateral chest	4.2±3.2 (2.7–5.9)	1.7±0.9 (1.1–2.0)	p<0.001
Upper abdomen	5.4±4.5 (3.5–8.0)	2.1±1.4 (1.6–3.0)	p<0.001
Lower abdomen	5.6±5.5 (3.6–9.1)	2.5±2.6 (1.4–4.0)	p<0.001
Lower back	6.4±4.9 (3.8–8.7)	4.7±3.6 (3.0–6.6)	p<0.01^3^
Hip	6.3±4.3 (4.5–8.8)	2.5±3.7 (1.7–5.4)	p<0.001
Front thigh	3.2±2.3 (2.5–4.8)	1.9±1.0 (1.4–2.4)	p<0.001
Lateral thigh	4.0±2.4 (3.1–5.5)	1.7±1.2 (1.3–2.5)	p<0.001
Rear thigh	3.5±2.4 (2.4–4.8)	1.7±1.6 (1.4–3.0)	p<0.001
Inner thigh	4.9±4.0 (3.8–7.8)	2.8±1.3 (2.1–3.4)	p<0.001
Calf	3.0±1.7 (2.2–3.9)	1.6±0.9 (1.3–2.2)	p<0.001
**Compartments (mm)**			
Arms^5^	7.5±4.0 (5.6–9.6)	3.7±2.0 (3.2–5.2)	p<0.001
Legs^6^	19.3±8.0 (15.8–23.8)	10.1±3.9 (8.5–12.4)	p<0.001
Abdomen^7^	24.9±18.8 (17.1–35.9)	12.1±8.7 (8.5–17.2)	p<0.001
Trunk^8^	14.6±10.9 (11.5–22.4)	6.5±2.2 (5.6–7.8)	p<0.001
Total SAT^9^	68.3±36.6 (52.9–89.5)	33.8±13.4 (26.5–39.9)	p<0.001
TBF%	15.4±4.7 (12.8–17.5)	10.2±2.9 (8.5–11.4)	p<0.001

Data is Median ± interquartile range (1^st^ to the 3^rd^ quartile).

1 By Mann-Whitney U test.

2 Not significant (p>0.05).

3 By t-test for independent samples.

4 SAT thickness of 15 body sites in mm.

5 Body sites biceps+triceps.

6 Body sites front thigh+lateral thigh+rear thigh+inner thigh+calf.

7 Body sites upper abdomen+lower abdomen+lower back+hip.

8 Body sites neck+upper back+lateral chest+front chest.

9 Body sites 1–15.

**Table 2:** Descriptive statistics of the two female groups.

Personal parameters	Female non-athletes (n = 21)	Female athletes (n = 21)	Significance of differences^1^
Age (y)	24.8±2.6 (23.6–26.2)	21.7±16.1 (17.1–33.2)	n.s.^2^
Height (m)	1.66±0.1 (1.62–1.71)	1.7±0.1 (1.64–1.73)	n.s.^3^
Weight (kg)	54.0±6.8 (52.0–58.8)	60.0±8.0 (55.0–63.0)	p<0.05
BMI (kg/m^2^)	19.9±1.0 (19.7–20.7)	20.8±2.1 (20.0–22.1)	p<0.05
**SAT-Top** 4			
Neck	5.8±3.6 (4.0–7.6)	2.4±2.1 (1.5–3.6)	p<0.001
Triceps	12.5±4.4 (9.9–14.3)	7.9±2.0 (7.0–9.0)	p<0.001
Biceps	5.3±3.6 (4.0–7.6)	3.2±2.0 (2.2–4.2)	p<0.001^3^
Upper back	4.9±2.6 (3.7–6.3)	2.3±1.4 (1.7–3.1)	p<0.001
Front chest	8.6±5.9 (4.4–10.3)	2.7±2.6 (1.8–4.4)	p<0.001
Lateral chest	6.3±5.4 (4.6–10.0)	2.2±3.3 (1.4–4.7)	p<0.001
Upper abdomen	7.4±5.2 (5.5–10.7)	3.8±5.1 (2.6–7.7)	p<0.01
Lower abdomen	10.2±6.9 (6.0–12.9)	6.3±4.7 (4.2–8.9)	n.s.^3^
Lower back	11.4±5.2 (8.6–13.8)	9.1±3.8 (7.2–11.0)	p<0.05^3^
Hip	8.5±6.6 (5.4–12.0)	7.1±7.7 (3.4–11.1)	n.s.^3^
Front thigh	10.3±3.5 (8.0–11.5)	6.9±3.6 (4.6–8.2)	p<0.001
Lateral thigh	10.4±2.8 (9.9–12.7)	8.0±3.4 (6.5–9.9)	p<0.01^3^
Rear thigh	7.2±1.9 (6.1–8.0)	5.8±2.7 (5.0–7.7)	n.s.^3^
Inner thigh	11.2±2.8 (9.8–12.6)	7.4±5.0 (5.4–10.4)	p<0.001^3^
Calf	6.3±2.3 (4.8–7.1)	3.5±2.8 (2.5–5.3)	p<0.001^3^
**Compartments (mm)**			
Arms^5^	17.4±5.2 (15.7–20.9)	11.1±2.3 (10.1–12.4)	p<0.001^3^
Legs^6^	46.6±9.1 (40.2–49.3)	30.9±16.1 (24.2–40.3)	p<0.001^3^
Abdomen^7^	40.5±21.4 (25.7–47.1)	26.5±23.2 (16.4–39.6)	p<0.05^3^
Trunk^8^	25.6±17.1 (17.1–34.2)	10.2±6.4 (7.8–14.2)	p<0.001
Total SAT^9^	133.7±48.6 (102.1–150.7)	78.8±42.1 (62.8–104.9)	p<0.001^3^
TBF%	30.2±5.5 (27.2–32.7)	26.9±4.7 (24.8–29.5)	p<0.01^3^

Data is Median ± interquartile range (1^st^ to the 3^rd^ quartile).

1 By Mann-Whitney U test.

2 Not significant (p>0.05).

3 By t-test for independent samples.

4 SAT thickness of 15 body sites in mm.

5 Body sites biceps+triceps.

6 Body sites front thigh+lateral thigh+rear thigh+inner thigh+calf.

7 Body sites upper abdomen+lower abdomen+lower back+hip.

8 Body sites neck+upper back+lateral chest+front chest.

9 Body sites 1–15.

The participants provided their written informed consent to the study after receiving a thorough explanation of the study and its requirements. For participants under the age of 18, two informed consents were provided, one for their caretaker and one for themselves, as required by the local ethics committee. The procedures used in this study were in accordance with the Declaration of Helsinki and were approved by the ethics committee of the medical university of Graz (IRB00002556) (EC-number 19-054 ex 07/08).

### Athletes

Twenty-three swimmers (8 females, 15 males) and 30 triathletes (13 females, 17 males) were recruited from triathlon and swimming clubs in Graz (Austria) and Christchurch (New Zealand). They were between the ages of 15 and 30 years with at least 3 years training experience. The training and competition frequency was at least 2 hr/day, 6 days/week. In a pre-test we investigated differences in body composition between swimmers and triathletes. We found no significant differences between the two groups, with the exception of the rear thigh measurement in women. Therefore we merged swimmers and triathletes to one group of athletes.

### Non-athletes

Non-athletes were recruited via an advertisement. The subjects of the non-athletic group were aged between 15 and 30 years, non-smokers, were currently taking no medication and performing no more than one hour of exercise per week.

### Measurement of SAT-Top

The optical Lipometer device was applied to measure the thickness of SAT in millimetres at 15 well-defined body sites distributed from neck to calf (see Figure S1). Measurements were performed on the right side of the body while subjects were in an upright standing position by a qualified technician. This set of measurement points defines the SAT-Top of each subject. The complete SAT-Top measurement cycle for one subject lasts about two minutes. The sensor head of the optical Lipometer device consists of a set of light emitting diodes as light sources and a photodetector. During measurement, the sensor head is held perpendicular to the selected body site. The diodes illuminate the SAT-layer and the photodetector measures the corresponding light intensities back-scattered. The resulting light pattern values of a measured body site were calculated to absolute SAT layer thickness (in mm) using computer tomography (CT) as the reference method. The level of agreement between CT and the Lipometer has been found to be very high (correlation coefficient of r = 0.99, with a regression line y = 0.97x+0.37, and no systematic deviation of the Lipometer measurements from the CT results [Bias = 0.0]) [18], [19]. In adults the reliability of the SAT-Top method produced coefficients of variation ranging from 1.9% (front chest) to 12.2% (rear thigh) [20].

### Statistics

Statistical calculations were performed by SPSS for Windows (version 16.0). Due to the distribution of the data the median, 1^st^Quartile (Q1), 3^rd^Quartile (Q3) and interquartile range (IQR = Q3–Q1) were used for the descriptive analysis of the various variables. The normal distribution of the variables was tested using the Shapiro-Wilk test and the Kolmogorov-Smirnov test. Differences in the distributions of variables between athletes and non-athlete controls was tested by a Student’s t-test for 2 independent samples (in case of normally distributed variables) and by a Mann-Whitney U-test for 2 independent samples (if variables were not normally distributed).

The 15 individual SAT-Top body sites listed in Table 1 and 2 have been described previously [21] (see Figure S1) and can be summed to estimate regional fat mass (e.g. arms [biceps+triceps], trunk [neck+upper back+lateral chest+front chest], abdomen [upper abdomen+lower abdomen+lower back+hip] and legs [front thigh+lateral thigh+rear thigh+inner thigh+calf]).

To give information about the total amount of subcutaneous fat in these two groups, all 15 SAT layer thicknesses were summed (Total SAT). Furthermore, TBF% was calculated by equations developed in a former study [22], using dual-energy x-ray absorptiometry (DXA) as reference method. To estimate Lipometer TBF% stepwise multiple regression analysis was applied, using the calculated DXA TBF% as dependent variable. Using the 15 Lipometer SAT thicknesses together with age, height, weight and BMI as independent variables provided the best estimations of Lipometer TBF% for both genders with strong correlations to DXA TBF% (R = 0.99 for males and R = 0.95 for females). The limits of agreement were −2.48% to +2.48% for males and −4.28% to +4.28% for females. For both genders a bias of 0.00% was determined [22].

**Figure 1:**
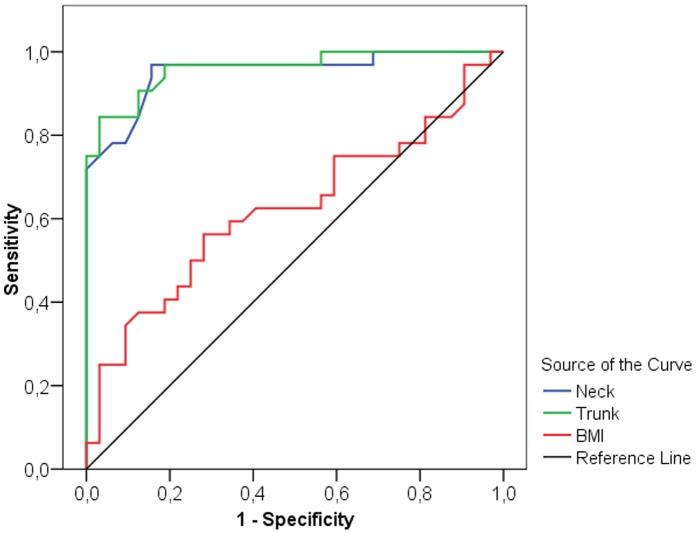
Receiver-operator characteristics (ROC) curve for BMI, neck measurement site and trunk compartment of men. The curve describes the association between sensitivity and specificity at different thresholds. ROC curves that approach the upper leftmost corner represent highly accurate classifiers.

**Figure 2:**
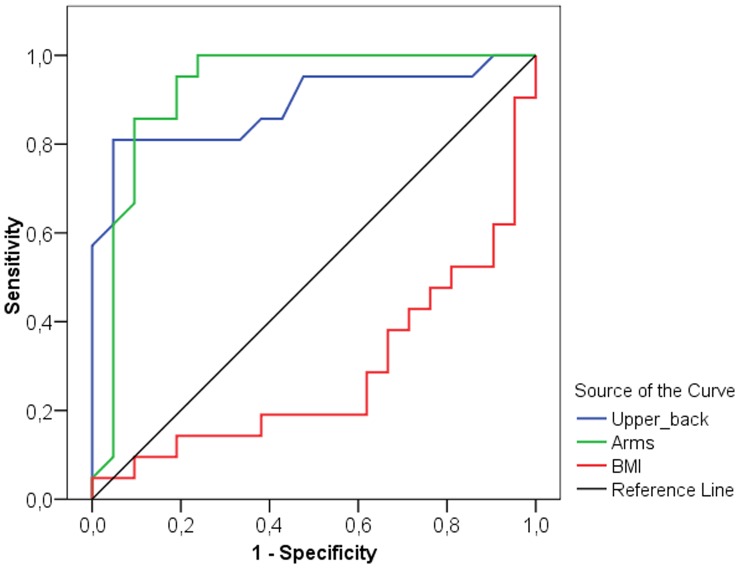
Receiver-operator characteristics (ROC) curve for BMI, upper back measurement site and arms compartment of women. The curve describes the association between sensitivity and specificity at different thresholds. ROC curves that approach the upper leftmost corner represent highly accurate classifiers.

The selectivity of measurement points was detected by receiver operating characteristic (ROC) curve analysis, which is a useful method for organizing classifiers and visualizing their performance [23], [24]. Two different a priori hypotheses were specified: that either smaller or larger parameter values are associated with stronger evidence of positivity ( = group of athletes). The area under the ROC curve is calculated and the result is expressed as an Area Index (AI). The higher sensitivity ( = the test’s ability to identify positive results) and specificity ( = the test’s ability to identify negative results), the more the ROC-Curve shifts into the upper left corner of the graph (high discriminating power) (see Figure 1) and the AI moves towards 1.0, consequently the selectivity between the groups is strong. Generally the AI can reach from 0.0 to 1.0 ( = strongest selectivity). If the curve is near the diagonal ( = AI 0.5) the selectivity is weak. An AI <0.5 shows that the a priori hypothesis should be changed (see BMI in Figure 2). In the ROC curve, the x coordinate represents the sensitivity and the y coordinate shows the specificity. The highest sensitivity and specificity were obtained at the optimal cut-off point estimated by the Youden index [25]. This optimal cut-off value provides the best discriminating power between the group of athletes and their controls, whereby smaller values are associated more strongly with the group of athletes.

## Results

Male athletes and non-athletes were similar in terms of age, height, weight and BMI, however, male athletes showed a 50.5% lower Total SAT thickness (33.8±13.4 mm) compared to male non-athletes (68.3±36.6 mm, p<0.001). All SAT layer thicknesses at the 15 body sites from neck to calf were significantly lower in the male athletes compared to the male non-athletes (Table 1). This was also the case for the additional variables (the four compartment measurements and TBF %).

Even though the female athletes had significantly higher BMI (p = 0.016) and weight (p = 0.011), their Total SAT thickness was 41.1% lower (78.8±42.1 mm) compared to their non-athlete counterparts (133.7±48.7 mm, p<0.001). SAT at all measured body sites, for all body compartments and TBF% was significant lower in the female athletes compared to the non-athletes except for the lower abdomen, hip and rear thigh ( = gynoid fat pattern) (Table 2).

ROC curves and the corresponding area indices were calculated for height, weight, BMI, TBF%, Total SAT, SAT-layer thicknesses at all 15 body sites and for the 4 compartments. The optimal cut-off values were analysed for body sites with a p-value of ≤0.05 and BMI. Results are presented in Table 3 and 4, and show the area indices for these variables one of the two assumptions that either small or large values provide stronger evidence for positivity ( = athletes).

**Table 3:** Area indices and optimal cut-off values obtained from ROC curve analysis for height, weight, BMI, 15 specified SAT-layers, 4 Compartments, Total SAT, and TBF% of 32 male athletes and 32 male non-athletes.

Personalparameters	Area index^1^		P	Optimal cutoff^2^	Sensitivity	Specificity	Correctly classifiedcases
	H_0_: small	H_0_: large		[mm]	[%]	[%]	
Height (m)		0.552	n.s.^3^				
Weight (kg)	0.527	–	n.s.				
BMI (kg/m^2^)	0.623	–	n.s.	21.9	56.3	71.9	64.1% (41 of 64)
TBF%	0.903	–	<0.001	11.5	78.1	93.8	85.9% (55 of 64)
Total SAT^9^	0.914	–	<0.001	51.7	93.8	78.1	85.9% (55 of 64)
**SAT-Top** 4							
Neck	0.952	–	<0.001	2.3	96.9	84.4	90.6% (58 of 64)
Triceps	0.853	–	<0.001	3.3	75.0	87.5	81.3% (52 of 64)
Biceps	0.901	–	<0.001	2.1	87.5	81.3	84.4% (54 of 64)
Upper back	0.929	–	<0.001	3.0	100.0	65.6	82.8% (53 of 64)
Front chest	0.889	–	<0.001	2.4	75.0	90.6	82.8% (53 of 64)
Lateral chest	0.914	–	<0.001	2.7	90.6	81.3	85.9% (55 of 64)
Upper abdomen	0.882	–	<0.001	4.2	96.9	68.8	82.8% (53 of 64)
Lower abdomen	0.844	–	<0.001	5.2	93.8	59.4	76.6% (49 of 64)
Lower back	0.698	–	<0.01	7.5	87.5	46.9	67.2% (43 of 64)
Hip	0.809	–	<0.001	4.2	68.8	81.3	75.0% (48 of 64)
Front thigh	0.831	–	<0.001	2.5	78.1	84.4	81.3% (52 of 64)
Lateral thigh	0.925	–	<0.001	2.8	87.5	87.5	87.5% (56 of 64)
Rear thigh	0.815	–	<0.001	2.1	56.3	93.8	75.0% (48 of 64)
Inner thigh	0.865	–	<0.001	3.7	81.3	81.3	81.3% (52 of 64)
Calf	0.821	–	<0.001	2.2	71.9	78.1	75.0% (48 of 64)
**Compartments**							
Arms^5^	0.907	–	<0.001	5.4	87.5	81.3	84.4% (54 of 64)
Trunk^6^	0.960	–	<0.001	15.5	84.4	96.9	90.6% (58 of 64)
Abdomen^7^	0.836	–	<0.001	19.8	84.4	75.0	79.7% (51 of 64)
Legs^8^	0.910	–	<0.001	8.2	93.8	78.1	85.9% (55 of 64)

1 There are two possible hypotheses (H_0_): that either small/large values provide stronger evidence for positivity.

2 Optimal cut-off value estimated by Youden-Index (Youden, 1950).

3 Not significant (p>0.05).

4 SAT thickness of 15 body sites in mm.

5 Body sites biceps+triceps.

6 Body sites front thigh+lateral thigh+rear thigh+inner thigh+calf.

7 Body sites upper abdomen+lower abdomen+lower back+hip.

8 Body sites neck+upper back+lateral chest+front chest.

9 Body sites 1–15.

**Table 4:** Area indices and optimal cut-off values obtained from ROC curve analysis for height, weight, BMI, 15 specified SAT-layers, 4 Compartments, Total SAT and TBF% of 21 female athletes and 21 female non-athletes.

Personalparameters	Area index^1^		P	Optimal cutoff^2^	Sensitivity	Specificity	Correctly classifiedcases
	H_0_: small	H_0_: large		[mm]	[%]	[%]	
Height (m)		0.595	n.s.^3^				
Weight (kg)		0.728	<0.05	66.0	95.2	9.5	52.4% (22 of 42)
BMI (kg/m^2^)		0.717	<0.05	18.8	4.8	100.0	52.4% (22 of 42)
TBF%	0.757	–	<0.01	30.5	100.0	47.6	73.8% (31 of 42)
Total SAT^9^	0.866	–	<0.001	83.6	61.9	100.0	81.0% (34 of 42)
**SAT-Top** 4							
Neck	0.901	–	<0.001	4.8	90.5	71.4	81.0% (34 of 42)
Triceps	0.908	–	<0.001	10.4	95.2	76.2	85.7% (36 of 42)
Biceps	0.853	–	<0.001	3.8	71.4	85.7	78.6% (33 of 42)
Upper back	0.888	–	<0.001	3.3	81.0	95.2	88.1% (37 of 42)
Front chest	0.881	–	<0.001	4.1	76.2	85.7	81.0% (34 of 42)
Lateral chest	0.866	–	<0.001	3.3	71.4	95.2	83.3% (35 of 42)
Upper abdomen	0.746	–	<0.01	4.7	57.1	85.7	71.4% (30 of 42)
Lower abdomen	0.663	–	n.s.				
Lower back	0.689	–	<0.05	11.6	85.7	47.6	66.7% (28 of 42)
Hip	0.616	–	n.s.				
Front thigh	0.859	–	<0.001	9.5	90.5	66.7	78.6% (33 of 42)
Lateral thigh	0.824	–	<0.001	9.1	71.4	95.2	83.3% (35 of 42)
Rear thigh	0.641	–	n.s.				
Inner thigh	0.842	–	<0.001	9.6	71.4	85.7	78.6% (33 of 42)
Calf	0.825	–	<0.001	5.6	85.7	66.7	76.2% (32 of 42)
**Compartments**							
Arms^5^	0.923	–	<0.001	15.9	100.0	76.2	88.1% (37 of 42)
Trunk^6^	0.909	–	<0.001	13.9	76.2	95.2	85.7% (36 of 42)
Abdomen^7^	0.707	–	<0.05	34.9	71.4	66.7	69.0% (29 of 42)
Legs^8^	0.854	–	<0.001	44.5	90.5	66.7	78.6% (33 of 42)

1 There are two possible hypotheses (H_0_): that either small/large values provide stronger evidence for positivity.

2 Optimal cut-off value estimated by Youden-Index (Youden. 1950).

3 Not significant (p>0.05).

4 SAT thickness of 15 body sites in mm.

5 Body sites biceps+triceps.

6 Body sites front thigh+lateral thigh+rear thigh+inner thigh+calf.

7 Body sites upper abdomen+lower abdomen+lower back+hip.

8 Body sites neck+upper back+lateral chest+front chest.

9 Body sites 1–15.

**Figure 3:**
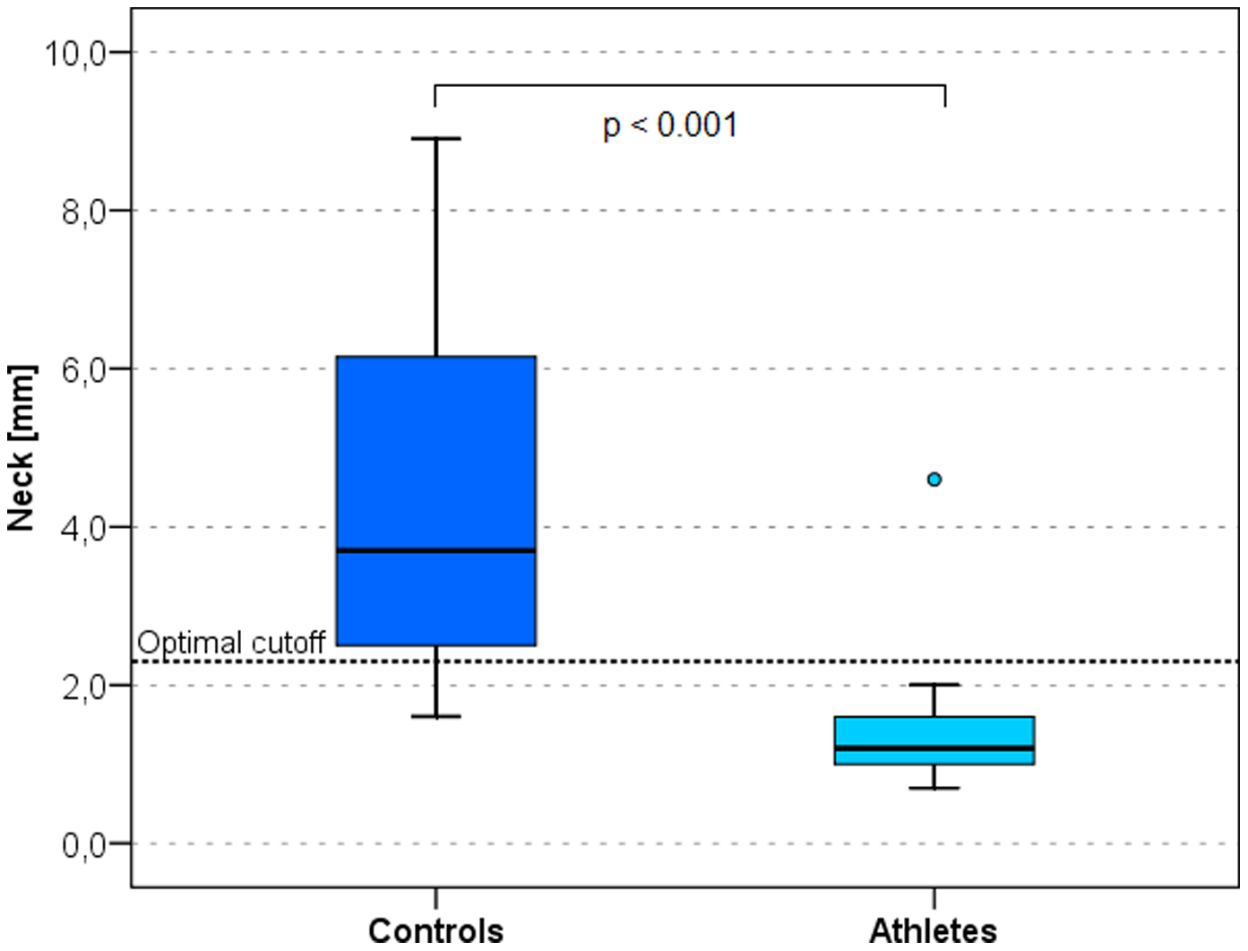
Box plots of the neck measurement site in athletes and controls. The neck is the body site with the highest discriminating power in men. The black horizontal lines represent the median, the box represents the 1^st^ and 3^rd^ quartile, the whiskers the 5^th^ and 95^th^ percentiles. Outliers are represented by dots. Optimal cutoff is marked by a dotted horizontal line.

**Figure 4:**
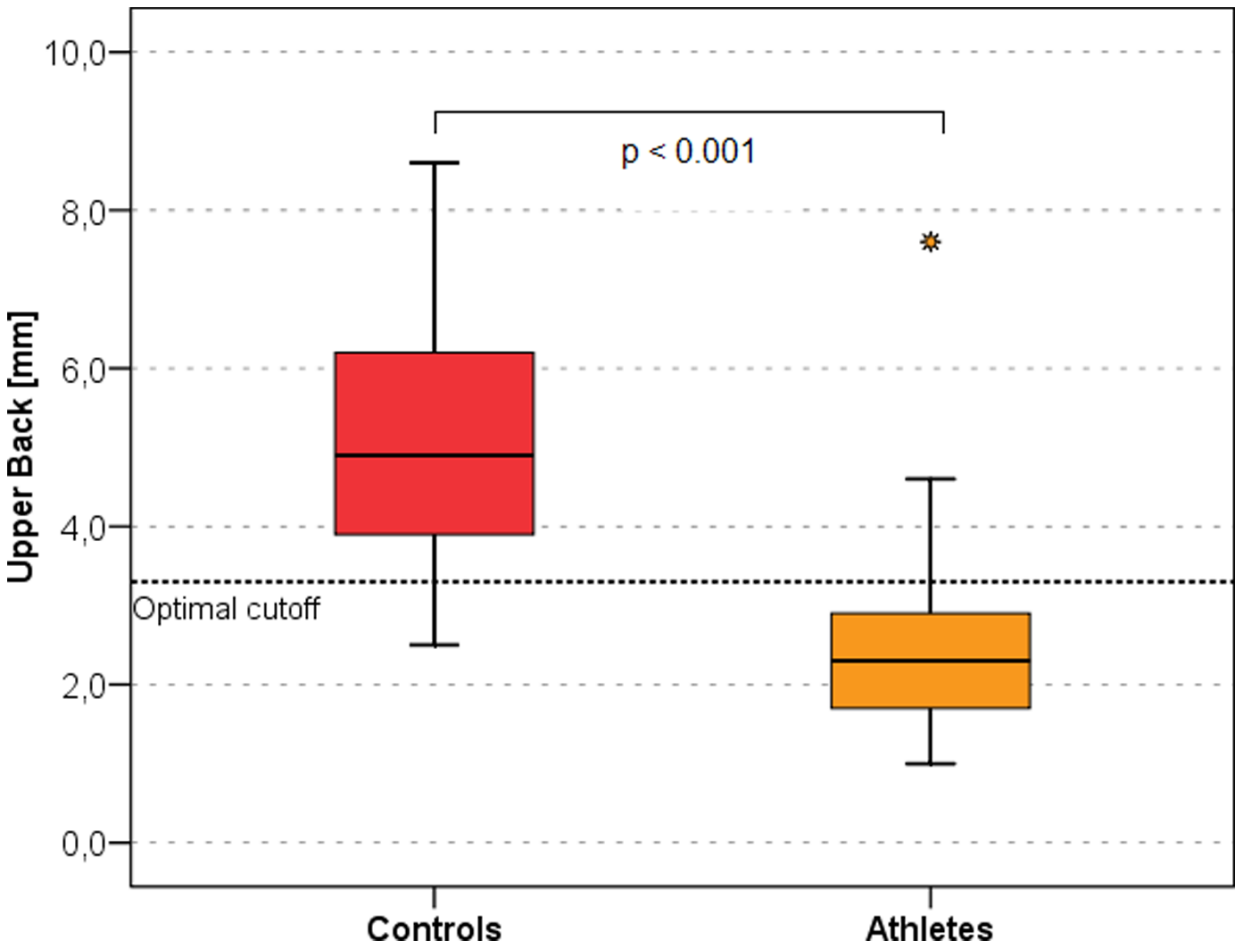
Box plots of the upper back measurement site in athletes and controls. This is the body site with the highest discriminating power in women. The black horizontal lines represent the median, the box represents the 1^st^ and 3^rd^ quartile, the whiskers the 5^th^ and 95^th^ percentiles. Outliers are represented by dots. Optimal cutoff is marked by a dotted horizontal line.

The best discriminators between male and female athletes and non-athletes are presented as ROC curves in Figure 1 and 2 respectively. In men the neck (Figure 3) measurement (AI = 0.952, sensitivity = 96.9%, specificity = 84.4%, optimal cut-off value 2.3 mm) and the trunk compartment (AI = 0.960, sensitivity = 84.4%, specificity = 96.9, optimal cut-off value 15.5 mm) provided the strongest discrimination power (90.6% [ = 58 of 64 of the subjects were correctly classified as athletes or controls]). The data showed no significant difference between the BMI of athletes and non-athletes (AI = 0.623, discrimination power: 64.1% [41 of 64 correctly classified subjects]) (Table 3). In women the upper back (Figure 4) measurement (AI = 0.888, sensitivity = 81.0%, specificity = 95.2%, optimal cut-off value 3.3 mm) and the arms compartment (AI = 0.923, sensitivity = 100.0%, specificity = 76.2%, optimal cut-off value 15.9 mm) provided the strongest discrimination power (88.1% [ = 37 of 42 correctly classified subjects]). Female athletes had significantly higher BMI, nevertheless the BMI AI was low (AI = 0.717, discrimination power: 52.4% [ = 22 of 42 correctly classified subjects]) (Table 4).

## Discussion

Our data shows that athletes and non-athletes of both sexes can be distinguished very clearly by their subcutaneous fat patterns. In spite of comparable BMI in the males, and even significantly higher BMI in the female athlete group, the measured SAT-Top values were significantly lower in the athletes compared to non-athletes in both groups. Male and female athletes showed approximately 50–60% lower Total SAT thickness compared to non-athletes. The ability of BMI to accurately reflect the amount of body fat across athletic and non-athletic populations has been assessed previously [6], [26]. Nevill et al. [6] report a 5–32% lower total skinfold thickness (measured by callipers) in male and 5–29% lower skinfold thickness in female athletes compared to their non-athletic controls. Furthermore, when Witt and Bush [26] examined the relationship between BMI and body fat in college athletes, the authors found that only 20% of women and 4% of men with BMI ≥25 kg/m^2^ were above the 85th percentile for skinfold measurements. Ode and colleagues [7] analysed the sensitivity, specificity, and predictive values for BMI as a measure of body fatness (measured via air displacement plethysmography) and found low sensitivity between BMI and body fat percentage for athletic populations.

Other investigators have examined the diagnostic ability of BMI in relation to TBF% in adults [11], [24], [27]–[32]. Our data as well as results of previous researchers show that BMI is a relatively poor indicator for the amount of body fat in young athletes and non-athletes. However, because of the lack of an established TBF% criterion for health status and the differences in study design, it is difficult to compare the results of our study with this previous research. Many of these studies used different methods for measuring TBF%, including DXA [27], [28], [30] skinfolds [29] and hydrodensitometry [11], [31], [32]. The different TBF% cut points used to identify over fatness included 25% [11], [28], 30% [29], [31], 33% [32], 35% [30] and 38% [27] for females, and either 20% [11], [28] or 25% [29], [30], [32] for males. With the exception of one study that assessed postmenopausal women [27], each study assessed both males and females. The majority of studies included young, middle-aged and older adults [11], [28]–[31], whereas an additional study focused primarily on young and middle-aged adults [32]. Within the postmenopausal women, BMI seemed to be a good diagnostic test for overfatness [27], however, the remaining research consistently indicated BMI had low sensitivity (0.06–0.60) and high specificity (0.86–1.0) as a measure of TBF% in both males and females [11], [28]–[32].

The results of our current study suggest that BMI is not an accurate predictor of overfatness in young athletes and non-athletes, indicated by the large differences between Lipometer-determined subcutaneous adipose tissue thicknesses and BMI values. Due to a larger muscle mass among the male and female athletes, BMI incorrectly classified normal fat athletes as overfat [33]. Therefore, our results indicate that the subcutaneous fat patterns are a better screening tool to characterize fatness and moreover for detailed fat distribution in physically active young non-athletes. This is particularly noteworthy, given that fatness is more influenced by sport (and therefore physical training) than is the patterning of fat [34]. Our results of the ROC curve analysis showed that in men the neck body site and the trunk compartment have the highest discrimination power between the groups of athletes and non-athletes (Figure 1). In women the highest discrimination power was achieved at the upper back body site, and the arms compartment (Figure 2). Also in previous published papers [19], [35] the neck body site became apparent as a good discriminator between normal weight healthy subjects and normal weight type-2 diabetes subjects. The above findings confirm the danger of using BMI in epidemiological studies, especially when a significant proportion of subjects come from a younger athletic population. When we monitor trends in fatness over time and between populations, a more valid method of assessing fatness is likely to be obtained using surface anthropometry such as the measurement of the neck or trunk compartment for males and the upper back or arms compartment for females with the Lipometer. Other methods to assess the body composition frequently lack precision and reproducibility (calliper techniques), entail the risk of radiation exposure (computed tomography (CT), dual energy X-ray absorptiometry (DXA)), depend on hydrational status (bioimpedance) are inconvenient and time-consuming for the patient (hydrodensitometry) and/or are expensive (nuclear magnetic resonance, CT, air displacement plethysmography) [36]. The Lipometer offers a new practical approach for body fat measurement.

### Perspectives

We have found that the subcutaneous fat patterns are a useful screening tool for (risk-) phenotypes in adults [19], [35], [37]–[40] and in children [41]. Whether the subcutaneous fat patterns are also useful for assessing risky phenotypes in adolescent and physically active young people is a subject of further investigation. However, to date, there is no adequate measurement system for a rapid, inexpensive, precise, portable, and safe determination of SAT distribution. SAT-Top as measured by the Lipometer meets these criteria. Based on the good discrimination results obtained from the present dataset, Lipometer SAT-Top measurements are likely to contribute to this interesting field in further studies.

## Supporting Information

Figure S1 **Specified body sites employed for LIPOMETER measurements of SAT thickness** [**21**] **.**
(TIF)
