# Supplementary figures and images for: Detecting Body Fat–A Weighty Problem BMI versus Subcutaneous Fat Patterns in Athletes and Non-Athletes
Source: PLoS One. 2013 Aug 26;8(8):e72002. doi: 10.1371/journal.pone.0072002 (PMC3753354; doi:10.1371/journal.pone.0072002)

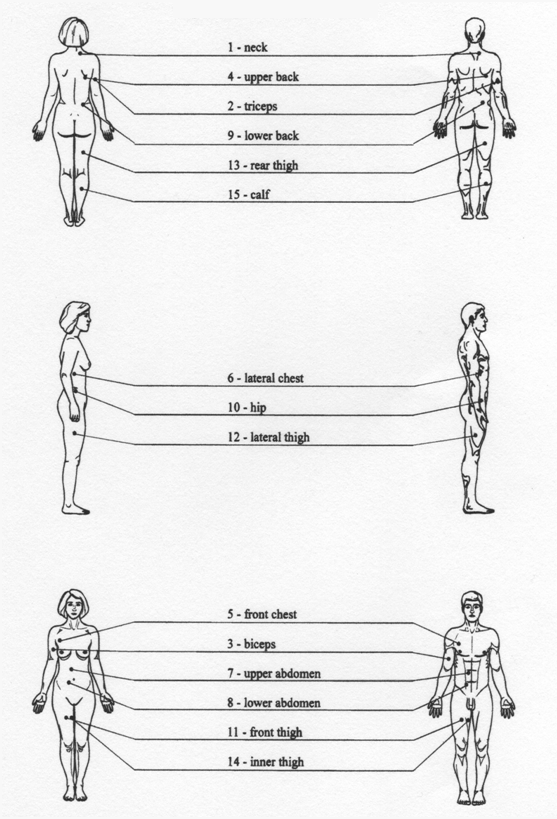

Supplement: Figure S1 — Specified body sites employed for LIPOMETER measurements of SAT thickness [21] . (TIF) [file pone.0072002.s001.tif]
